# Parametric Urbanism and Environment Optimization: Toward a Quality Environmental Urban Morphology

**DOI:** 10.3390/ijerph18073558

**Published:** 2021-03-30

**Authors:** Yingyi Zhang, Chang Liu

**Affiliations:** 1 School of Architecture and Urban Planning, Beijing University of Civil Engineering and Architecture, Beijing 100044, China; zyy2012arch@gmail.com; 2 Tsinghua Shenzhen International Graduate School, Tsinghua University, Shenzhen 518055, China

**Keywords:** parametric urbanism, environmental optimization, integrated approaches, urban morphology, quality environment

## Abstract

Parametric thinking has found wide acceptance in both the building industry and environmental sciences. In the context of environmental urban morphology however, parametric thinking has been neglected. This paper critically assesses environmental optimization with a focus on parametric urbanism. The analysis addresses two research questions: “Can parametric thinking and its associated approaches facilitate an environmental urban morphology?” and “If yes, can parametric urbanism support environment optimization in complex urban areas?” Methodologies include a case study in Beijing, qualitative and quantitative analysis, parametric modeling, and environmental simulation. Results indicate that parametric techniques can effectively simulate environmental urban morphology by generating parametric models. These models provide a rational foundation for schematic decision-making about optimizing environments in urban development. Findings include a critique of parametric thinking as applied to city environments and insights about the potential uses of parametric techniques to support quality environmental urban morphology.

## 1. Introduction

From the 1980s, parametric thinking has evolved the fields of architecture, construction, and engineering. The computer-aided tools of parametric thinking have redefined the relationship between architectural design and shaping urban morphology. It has also impacted human-environment generation and regeneration. To some extent, the built environment has become a product of computable processes and integrated approaches. Parametric thinking enables tangible and intangible systems to be incorporated into design proposals that are removed from computational tool specificity. It asks architects to start with design parameters, rather than preconceived or predetermined design solutions [1]. According to Kolarevic (2005), parameterization has its own rules, methods, and features. It rejects static or rigid solutions, instead considering variables as lively, dynamic, and mutable elements of a design system. A parametric approach accommodates the need for recurring adaptation across design project processes [2].

Parametric thinking has been implemented in urban design practices. Pioneer architects and urban designers such as Zaha Hadid and Patrick Schumacher have carried out parametric design practices on multiple urban scales. Their design schemes for the Kartal Masterplan and Singapore One-North, for example, revolutionized people’s cognition of urban space. (Yes, the avant-garde forms did result in some controversy.) Consequently, recognition of the potential usage of parametric tools intensified technological investments in this area over the last decade [3]. Parametric urbanism arises from these practices. As per Gu (2018), parametric modeling originates from generative design and analysis. It is an integrated approach based on rules or algorithms (e.g., in generative grammars or evolutionary systems) [4]. Driven by the features of cities as hybrid territories, the methodologies of urbanism take advantage of parametric tools to optimize urban-scale design models and manipulate the dynamics of urban morphology. As Nagy (2009) mentioned, from the first experiments using parametric tools in architectural design processes, it was clear that these tools could similarly benefit urban design projects, including higher-scale urban cases [5].

Parametric urbanism is based on parametric design systems in which the parameters of a given object are declared but not that object’s performance. It offers an opportunity for architects and urban designers to optimize urban forms and spatial construction through the lens of parameters. Parameters work as variables within a system that can control design performance from the minutiae of architectural detail to large-scale developments. For example, the width and length of a street can delineate parameters for improving a street’s form and function. Urban designers can modulate data values with parametric modeling software, such as Grasshopper 3D (Robert McNeel and Associates. Seattle, Washington, DC, USA). They might make the street more pedestrian friendly or more convenient for automobiles according to their functional analysis of a specific street. In the case of multiple street networks on a large scale, parametric software can analyze complex parameters intuitionally. The results of this analysis can then help architects and urban designers reveal urban environment ideals. As Leach (2009) declares, design lead by classic geometric shapes can prove inflexible when adaptivity is essential [6]. Parametric urbanism, conversely, frees design solutions from directions led by axial forces or the position of urban objects. Design is instead guided by the distribution and density of the constructed urban fabric, by sensitivity to deformations within territories, and by the development of compositional gradients (such as the transformations across the heights of buildings) [3]. Parametric urbanism encompasses an inherent appreciation of the spatial individualities that define a city.

Environmental optimization has become more and more the essential driver of urbanism. Optimization is a search process for a solution addressing the special conditions of a specific problem [7]. Environmental optimization in urbanism refers to creating, or recreating, livable and sustainable urban areas through optimal schema. Numerous environmental challenges, such as microclimates in high-density cities, affect urban growth directions. Masterplans tend to be oriented toward optimizing physical urban morphology to create environmentally friendly cities by achieving human comfort and reducing fossil fuel energy demands [8]. Therefore, urbanism processes require significant consideration of environmental influences. As Salat and Adhitya (2009) say, urban morphology is the specific texture of a city informed by such parameters as building forms, street patterns, and population density [9]. The tangible forms of urban morphology deal with the physical composition of an urban environment and the spatial structure and form of various activities. Urban morphology has potential to embrace not simply a two and three-dimensional methodology that studies and characterizes the physical evolution of cities but one that facilitates a fuller understanding of intangible forms, such as the functional and socioeconomic evolution; it also has a contribution to make in the sustainable evolution and development of cities worldwide [10]. Form-based code has been working as an approach in a plenty of urban projects for morphology shaping. It creates or recreates a specific urban morphology primarily by controlling physical form, with a reduced focus on land use, through city or country regulation [11]. However, form-based code is still paper based. Its code generation can be inefficient, especially during the amendment process in large-scale projects [12,13]. Hence the parametric techniques are introduced into the morphological shaping processes to improve efficiency. To analyze the use of parametric techniques in environmental optimization, this paper limits the research range of urbanism to its physical representation and urban morphology, rather than population, economy, or society. The goal of the study is to use parametric techniques to facilitate environmental optimization in a complex urban context to achieve a quality environmental urban morphology. Research question is as follows: Can parametric thinking and approaches facilitate environmental urban morphology? If yes, can parametric urbanism support environmental optimization in complex urban areas? Accordingly, the potentiality of parametric techniques as applied to environmental urban morphology is analyzed, assessing the benefits that parametric urbanism may have in creating quality living environments.

## 2. Materials and Methods

**Figure 1:**
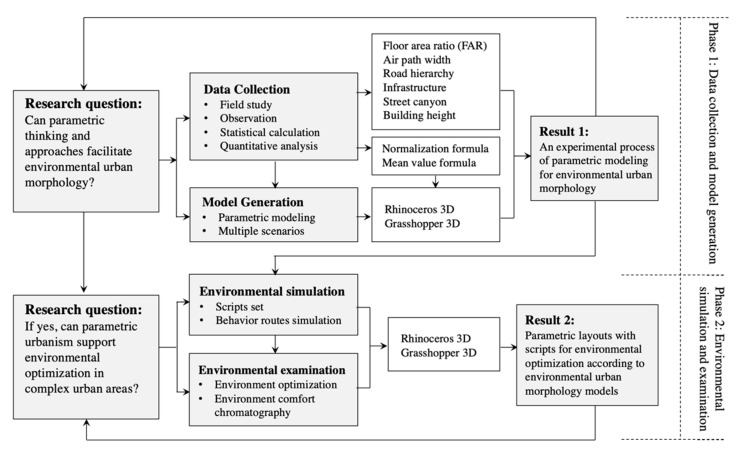
Method framework.

The method framework incorporates two phases (Figure 1). The first phase consists of data collection and model generation. Data collection works to understand the physical morphology of the research areas and prepare the geographic data to generate models. Both physical and geographic data are imported into the parametric modeling software. Parametric models perceive the existing urban morphology in a three-dimensional manner. Methods include field study, statistical calculation, quantitative analysis, and parametric modeling. The result is an experimental process of parametric modeling for analyzing environmental urban morphology. This phase targets the first research question about parametric thinking and approaches can facilitate environmental urban morphology or not. The second phase encompasses environmental simulation and examination. Environmental simulation presents the tendency of people’s routing behavior according to the three-dimensional models generated in the last phase. The result indicates which areas attract people to take part in outdoor activates or enjoy the environmental comfort. It also provides a foundation for environmental examination. In the examination part, the unattractive outdoor areas can be optimized by adjusting the parameters of the three-dimensional models. Methods consist of parametric modeling, simulation, and qualitative analysis. The result is parametric layouts with scripts. Urban designers and environmental analysts can use the parametric layouts to optimize the environmental urban morphology according to the realistic demands. This phase contributes to answering the second research question about whether parametric urbanism can support environmental optimization or not in complex urban areas.

### 2.1. Data Collection

#### 2.1.1. Material Data Types

Urban morphology comprises both tangible and intangible forms. The former is composed primarily of the urbanscape’s appearance: the geometric form of its land, the various patterns of its functions, the layout of its buildings, etc. The latter involves cultural, social, and other intangible elements. This paper focuses on the tangible form of the material environment. Based on the figure–ground theory, the elements of the material environment include two primary categories: The figure category includes examples such as building lots, infrastructure, and outdoor barriers. The ground category incorporates green areas, open space, and squares [14]. Data collected in figure and ground categories encompass three aspects: building complexes, street networks, and open space.

Environmental forces affect human comfort in a built environment, especially in high-density cities. Urban morphology shapes or reshapes environments, and vice versa. Whether generating an entirely new urban area or inserting an urban section in an existing area, the environment is inescapably changed. Six variables related to environmental urban morphology work to analyze environmental optimization in parametric urbanism: floor area ratio (FAR), air path width, road hierarchy, infrastructure, street canyon, and building height (Table 1). The six variables are chosen because they are related to environmental urban morphology. They are commonly used to define urban forms in urban environmental research and morphological shaping projects, such as the Miami 21 Code, SmartCode Montgomery, etc. Each variable has a specific measurement manner. For example, FAR is a ratio, and air path width is measured in meters. The normalization method works to achieve dimensionless values. It converts the original value to data of comparable magnitudes. The normalized data can be directly embedded into the parametric modeling system. The average value of each area indicates the artificial features of each area. If the value is high, it means the area has artificial features. People may cluster in this area. The outdoor space may accommodate various outdoor activities, traffic flow, etc. During the simulation phase, if the urban area with high average normalized data shows unattractive environmental urban morphology, it means urban designers and environmental analysts need to reshape the morphology to meet functional requirements.

1. FAR
  FAR acts as a quantized variable to represent the physical environment. It is the measurement of a building’s floor area in relation to the size of the lot/parcel that the building is located on. FAR value reflects building density, urban land usage, and the health of the urban atmosphere.
2. Air path width
  Air path width reflects the wind flow corridors between buildings or constructions. As per Ng (2010), the width of the street must be wider than the height of buildings; otherwise, skimming flow will dominate [16].
3. Road hierarchy
  Road hierarchy helps to estimate vehicle flow. A high-grade road suggests high levels of auto-transportation. This may result in high carbon emissions and pollution.
4. Infrastructure
  Infrastructure describes municipal service facilities. To a certain extent, it indicates the population gathering. Ample infrastructure reflects high-level artificial activities and clusters of communities.
5. Street canyon
  Street canyon is defined by the width of a specific street and the building height along that street. The ratio of depth-to-width impacts natural ventilation. The higher this ratio, the weaker the wind at the ground level [16].
6. Building height
  Building height reflects the skylines from a morphology perspective, as well as city ventilation from an environmental one. Taller buildings catch the wind passing through the city and downwash it into the city.

#### 2.1.2. Geographic Data Types

**Figure 2:**
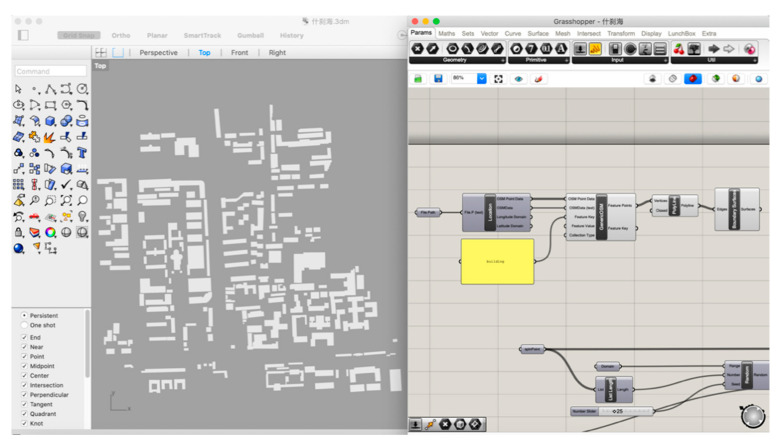
Constraint node data on the parametric modeling platform.

An urban zone in Shichahai, Beijing, forms the case study for data collection. The chosen zone is located in the central city of Beijing, adjacent to the main axis of the old city region. It consists of both conventional residential areas and modern business areas with a relatively high-density morphology. Shichahai fits the research target of using parametric instruments to analyze environmental urban morphology in a dense urban zone. Simulation and examination results will indicate the environmental quality in later experimental phases. The geographical information is obtained through an OpenStreetMap (OSM) format file. OSM is an editable map of the world supported by the OpenStreetMap Foundation, a non-profit organization registered in England and Wales. It has been enabled by portable satellite navigation devices [17]. OSM files contain a multitude of points of interest, buildings, natural features, and land-use information, as well as coastlines and administrative boundaries [18]. This paper uses building clusters from OSM to generate the figure–ground base map. The constraint node data of buildings are grabbed from OSM Online. We used Grasshopper 3D with its plug-in, Elk, a parametric modeling software, which translates the OSM file into coordinate points (Figure 2).

#### 2.1.3. Data Normalization

Besides the constraint node data, the six material data types, including FAR, air path width, road hierarchy, infrastructure, street canyon, and building height, were collected through field study. The research area consists of eight parts according to local neighborhood divisions. Each neighborhood has official edges. These neighborhoods are divided by streets and roads. The research team conducted the physical data collection for each part. Collected data require statistical analysis to achieve dimensionless indicators. The normalization methods convert the original data to data of comparable magnitudes. After reducing dependency and redundancy, the original data can be grouped and classified directly—despite being recorded with differing measurements and ranges [19].

Data normalization can include both data-oriented processing and dimensionless processing. The former aims to change data properties; the latter to compare variables with different natures. This paper uses dimensionless processing to normalize data without changing its properties. Statistical approaches include three representative normalizing methods: Z-score normalization, decimal scaling normalization, and min–max normalization. Z-score is appropriate for situations in which maximum and minimum values cannot be conformed but mean value and standard deviation are conformed. Decimal scaling suits cases where data contain many decimals. They are both unsuitable for this research. Consequently, min–max normalization was used to normalize the original data collected for this research.

As per Formulas (1) and (2), the normalization process transforms data and their average values into a closed interval [0,1]. Each part of the case study urban zone has a series of normalized data that reflects the parameters of its environmental urban morphology. The normalized data can be input into the parametric modeling process without being impacted by variances in data collection measurement and range. Data normalization results are presented in Table 2.

(1) $$\mathrm{Normalized}\;\left(\mathrm{ei}\right)=\frac{\mathrm{ei}-\mathrm{Emin}}{\mathrm{Emax}-\mathrm{Emin}}$$

where ei = the original data. Emin = the minimum value of E range. Emax = the maximum value of E range. If Emax is equal to Emin, the normalization result is 0.5.

(2) $$x=\frac{\sum_{k=1}^{n}\mathrm{Xk}}{n}$$

where x = mean value of normalized data. X_k_ = X_1_. n = the number of the variables.

### 2.2. Model Generation

#### 2.2.1. Algorithm Logic

The core of parametric modeling is the algorithm logic. An algorithm is expressed within a finite amount of space and time and in a well-defined formal language for calculating a function in mathematics and computer science [20]. The algorithm proceeds by successive subtractions in two loops: if the test B ≥ A yields “yes” (or true) (more accurately the number b in location B is greater than or equal to the number a in location A) then, the algorithm specifies B ←B − A (meaning the number b − a replaces the old b). Similarly, if A > B, then A ← A − B. The process terminates when (the contents of) B is 0, yielding the greatest common divisor in A [21,22]. The algorithm starts from an initial state and initial input and describes a computation that, when executed, proceeds through a finite number of well-defined successive states, eventually producing “output” and terminating [23].

#### 2.2.2. Software and Scripts

A series of software has been applied to generate parametric models. According to Schumacher (2009), parametric design works as a style rooted in digital animation techniques, and its latest refinements are based on advanced parametric design systems and scripting methods [24]. This paper utilizes Grasshopper 3D and Rhinoceros 3D (Robert McNeel and Associates. Seattle, Washington, USA) to edit scripts and build models. Grasshopper 3D and Rhinoceros 3D rely on algorithmic logic to create geometry with a visual programming language interface [25]. They can effectively present rational models on canvas by editing components and parameter relations. Altering parameters causes changes to propagate throughout all functions and the geometry to be redrawn [26].

**Figure 3:**
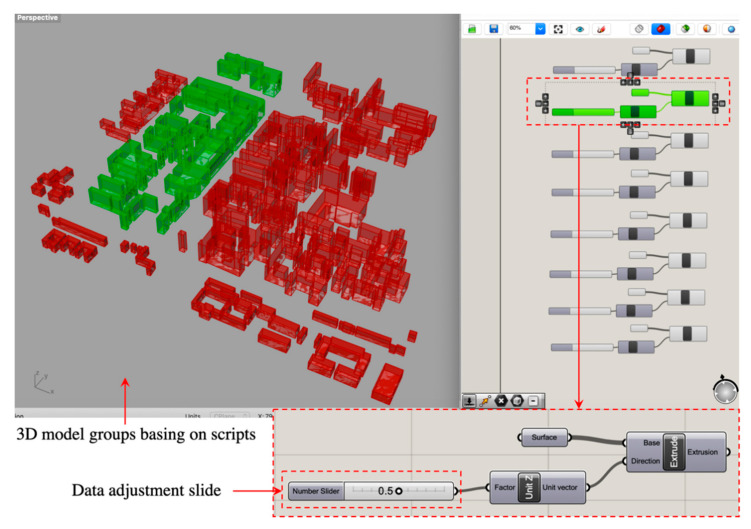
Scripts on Grasshopper 3D.

During the data collection phase, the base map is imported into Grasshopper 3D. In the script section, the existing built environment and proposed schemes are deconstructed into parameters, components, and mathematical rules. Figure 3 presents the relationships between scripts and parametric models. The scripts consist of a series of script units. Each unit contains parameter data adjustment slides. According to the data normalization result, data values can be directly set by these slides. A parametric model of the existing built urban morphology can then be presented on the canvas of Rhinoceros 3D. A variety of schemes can also be presented in real time by changing the data adjustment slides.

#### 2.2.3. Parametric Modeling

The parametric modeling process relies on the capacity to alter geometrical shapes when dimension values are changed. Programming codes, such as scripts, define the dimensions of the generated models. Parametric models with various parameter values and morphological characteristics are more flexible than those produced by conventional methods. Conventional methods normally offer designers two-dimensional drafts from which to obtain non-parametric models. Changing schemes therefore requires considerable time and human resources. Parametric methods, however, enable the modeling process to be synchronous with parameter manipulation. When multiple scenarios are required, parameter manipulation provides real-time, updated models by manipulating scripts in Grasshopper 3D [18]. Numerous urban morphology layouts can be automatically generated by computerized calculation. Three-dimensional modeling provides an approach for urban designers and environmental analysts to shape or reshape environmental urban morphology toward a quality urban environment. The experiment is based on the current existing morphology, but the generated models are dynamic. When changing the parameters of the models, various scenarios and public space evaluations will display in real-time. After a few rounds of modification, designers can select the most well-suited of the resulting models for further research or practice. The adjusted parameters and models indicate the schema on the basis of parametric scripts of the Shichahai urban zone of Beijing.

### 2.3. Environmental Simulation and Examination

Varying models and parameters of urban morphology make it possible to determine the environmental comfort of different urban textures and their impacts on people’s behaviors [27]. In dense cities, environmental urban morphology remains an open problem in terms of shaping urban space and improving outdoor activities. People prefer outdoor spaces with plenty of sunshine, a pleasant space scale, open sight, and moderate winds. However, the perfect outdoor space is difficult to provide in high-density cities. For example, dense high-rise buildings lead to poor solar illuminance in some streets, although they may reduce the force of air currents in windy seasons. Sparse building clusters contribute better lighting conditions but can create strong winds—especially in cold winters. Individual elements such as wind or light alone are not enough to support environmental optimization. Therefore, instead of relying solely on the thermal or windy effect, this paper uses comprehensive parameters to both simulate and examine environmental quality. A set of scripts simulate and measure environmental urban morphology through the prediction of human behavior trends. The simulating process is based on outdoor morphological shaping rather than observing a hundred people to record how they spend their time, where they go, and how long they stay in outdoor areas. The simulation is based on the outdoor morphological shaping. Parametric modeling software calculate the moving route tendency rather than a specific individual’s real moving route. This assists urban designers and environment analysts to take measures to improve the quality of unattractive areas. The simulation and measurement are established upon the models of the Shichahai urban zone provided in the former phase.

**Figure 4:**
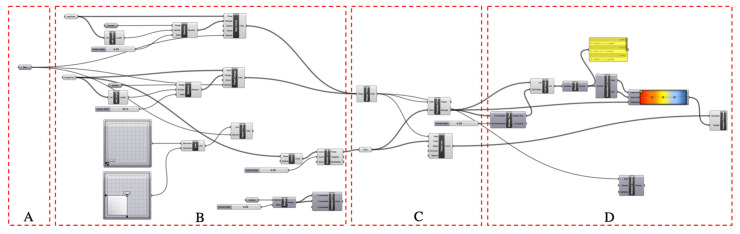
Script set for environmental optimization.

The scripts set for environmental optimization comprise four sections (Figure 4). Section A limits the simulation range to coincide with the field study boundary of Shichahai. Section B sets the coordinate points of attraction and interference areas. Attraction areas include downwind direction zones, people’s movement and destinations, and southern zones with relatively sufficient sunlight. Interference areas include the natural water system that cannot be crossed and the northern zones with insufficient sunlight. Section C runs scripts to generate field lines. Section D presents the degree of environmental comfort with chromatography.

## 3. Results and Discussion

### 3.1. A Parametric Modeling System for Environmental Urban Morphology

This paper analyzes parametric thinking and techniques for facilitating environmental morphology in an urban context. An experimental parametric modeling system is generated to support quality environmental urban morphology in terms of environmental comfort and urban morphology pleasantness. Environmental comfort has become an essential driver in urbanism processes. Urban morphology, a major physical representation of urbanism, reflects the quality of the built environment. Existing urbanism schemes rely heavily on manual methods. The experimental parametric modeling system applies parametric techniques to environmental urban morphology simulation and evaluation. This system facilities morphological shaping processes by providing rational calculation results, visualizing three-dimensional models, and presenting built-environment consequences.

**Figure 5:**
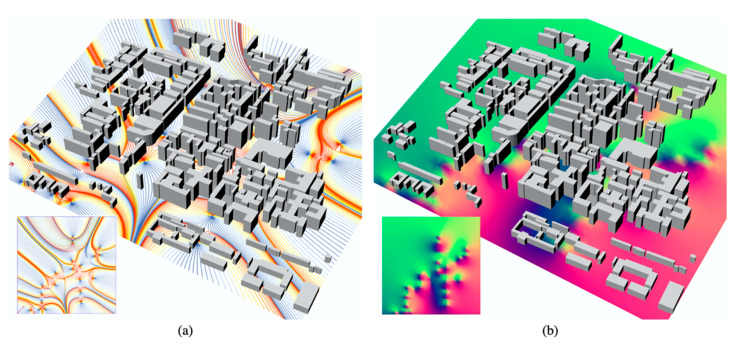
Environmental optimization simulation in Rhinoceros 3D and Grasshopper 3D. Figure (**a**) indicates the field line simulation results. The more field lines closer to blue, the more people possibly reject that outdoor environment. Figure (**b**) indicates a measurement of environmental optimization. Areas that tend to red shows relatively pleasant environmental conditions.

The computer calculates the urban environment situation automatically and presents the results on a Rhinoceros 3D canvas when the scripts sets are running in Grasshopper 3D. Take the model of Shichahai’s urban morphology as an example: The model indicates the degree of environmental attractiveness (Figure 5). It reflects whether people are willing to choose a specific place for outdoor activities. The more field lines closer to red, the more willing people are to exercise, communicate, or stay. The more field lines closer to blue, the more people reject that outdoor environment (Figure 5a). Contributing factors may include morphology scale and environmental wind or light situations. Another model presents a measurement of environmental optimization (Figure 5b). Areas that tend toward red indicate relatively pleasant environmental conditions. These areas are concentrated on the southeast part, closed to water banks and greenery. Areas that tend toward blue indicate that environmental conditions can be improved. The environment might be optimized by adjusting building heights, building enclosures, acquiring small-scale open spaces, etc., to support optimization in the environmental urban morphology context. In real urban projects, changing the modeling range may lead to different simulation results. The range could be with a radius of various numbers. It depends on how urban designers and environmental analysts use it or what the design or research requirements are. The Shichahai model is an example to run the scripts. The experiment aims to offer a universal simulation system rather than only be used in one specific urban zone.

The parametric modeling system has its roots in algorithmic logic and relies on the establishment of human–computer interaction. Human–computer interaction tools provide an opportunity to join algorithmic logic with parametric modeling. Grasshopper 3D, for example, translates algorithmic logic to visible command components. People—including those with limited coding knowledge—can manipulate components to achieve corresponding models.

Human–computer interaction modes work across the whole process of parametric modeling for environmental urban morphology. During the pre-modeling stage, general urban morphology characteristics and development trends are determined through preliminary field study and data integration. Manual methods constitute the foundation of parametric modeling. After that, geographic information and data integration results are both input into parametric modeling software. According to the manual edition of parameter types and their correlations, a computer automatically calculates models that meet the manual edition. These models can represent the existing environmental urban morphology but, more importantly, generate infinite models by adjusting parameter values or correlations. They can depict various scenarios, morphology characteristics, and socioenvironments to generate urban ideals. Comparing with conventional paper-based or form-based approaches, the parametric modeling system offers a visualized modeling interface to manipulate parameters and achieve interactive performance feedback at the early stage of morphological shaping. Therefore, the parametric modeling system facilities environmental urban morphology in the aspects of:

- Efficiency
  The system synchronizes human thoughts with parametric models—avoiding the investment of time and human resources to generate manual models.
- Flexibility
  It has the capability to support the analysis of multiple morphology types by adjusting parameter values or data. Parametric modeling systems have potentially wide-ranging applicability, from parametric urbanism to environmental sustainability.
- Visualization
  Urbanism strategies become more foreseeable and visible. Strategies no longer exist only in text descriptions and static images. Parametric models provide vivid pictures for planners, urban designers, environmentalists, architects, municipalities, developers, and neighborhoods and communities. The models also can connect with networks for public participation toward a rational and practicable environmental urban morphology scheme.

Although the parametric modeling system experiment explored in this paper can facilitate the generation of environmental urban morphology, it is not a panacea resolving all issues of urbanism. This paper simulates urban morphology with a few parameters related only to physical form. However, many more factors and parameters impact real life. For example, intangible factors such as cultural backgrounds, social development, and economic influences have not been considered here. Overemphasizing parametric techniques in physical environments may limit the success of addressing urban challenges.

### 3.2. An Alternative Approach to Environment Optimization

Urbanism requires consideration of environmental impacts. Current science provides processes for evaluating indices such as the potential for global warming, acid rain, photochemicals, etc. However, environments—particularly those with complex urban textures—require consideration alongside other concerns, such as habitability, walkability, and sustainability. Natural daylight, wind, etc., are not in the range of urban morphology shaping. This paper introduces parametric simulation and evaluation to support environmental optimization in urbanism in an urban morphology context. The simulation and evaluation results indicate that parametric approaches can partly reflect people’s behavior trend and route tendency. Urban designers and environmental analysts decide where the unattractive morphology is and which areas should be improved. Pleasantness of an environment is a broad term. The simulation and examination process analyses a small part—morphological evaluation—to see whether the environment needs to be improved or not. The results clearly contribute to environmental optimization and parametric urbanism.

Parametric tools are applied in living comfort for environmental optimization. This does not mean that parametric tools create a perfect system for predicting people’s behavior and their understanding or appreciation of environmental comfort. Human behavior is subject to individual differences, and it varies with external effects. The simulated constraint lines indicate human movement only as it corresponds to the parametric model. Other factors that motivate people’s behavior, such as the price of real estate or the commercial quality of a shopping mall, are not reflected through parametric approaches. Additionally, the chromatography of environmental comfort in parametric models is calculated by computer processes. No clear evidence proves absolute accuracy. In reality, environmental comfort comprises multiple parameters such as solar altitude, season and temperature, air pressure, and wind speed and direction on a specific day. They are not included in the modeling scripts. Former researchers normally use sensors, e.g., light sensors, smoke detectors, or air quality sensors, to collect data on natural conditions. These data types are different from those used in this research. This research analyzes environment quality from the perspective of urban morphology. It is about physical forms, including building height, street width, FAR, etc. Parametric techniques can assist in environment quality improvement through modeling, simulating, and evaluating the environmental urban morphology, but it is not a panacea. It is not the sole solution for addressing all urban environmental issues. Overemphasizing parametric modeling results may create inaccuracies in decision-making. There is no evidence demonstrating that parametric models provide better solutions than conventional manual works. The parametric modeling system has its features of effective, automatic, and flexible simulation. It is an alternative to assist decision-making rather than totally replacing manual methods. As a result, the parametric approach to environmental optimization may work best at the conceptual stage of urbanism, where it provides parameter- and data-based simulation and evaluation.

## 4. Conclusions

This paper critically analyzes the principles and mechanisms of parametric technologies. It generates an experimental parametric modeling system to demonstrate the potential application of that technology in environmental optimization to achieve a quality environmental urban morphology. Earlier environment research endeavors relied largely on collecting data and samples to evaluate environments. This paper proposes an alternative method. Parametric models of environmental urban morphology are generated to depict urban situations and trends. Parametric urbanism can include environmental optimization from the outset.

The results answer the research questions as follows: Parametric thinking and approaches can indeed facilitate environmental urban morphology. Parametric urbanism does have the capability to support environmental optimization in complex urban areas. However, it does not necessarily result in perfect environmental optimization strategies. The parametric modeling system in this paper can work best at the conceptual stage of urban morphology shaping and environmental optimization analysis. The parametric simulation and evaluation can act as a reference for the study of urbanism.

In the longer term, parametric methodologies will bring more convenient and dynamic support for urban environmental development. However, there is no clear evidence that it can produce better morphology design solutions than manual methods yet. The implementation of parametric technology requires further research.

## Figures and Tables

**Table 1:** Data types and descriptions.

Urban Morphology Aspect	Data Type	Urban Morphology Aspect	Data Type	Urban Morphology Aspect		Data Type
Building Complex		FAR	Street Networks	Air Path Width (m)	Open Space	Infrastructure
		Building Height (m)		Road Hierarchy		Street Canyon
**Road Hierarchy Grade**						
**Grade**		**Speed (km/h)**	**Lane**	**Motorway Width (m)**	**Total Width (m)**	**Median Strip**
1		<30	≥2	3.50	16–30	N/A
2		30–40	≥2	3.50	20–40	N/A
3		40–60	≥4	3.50	30–60	√
4		60–80	≥4	3.75	40–70	√
The above grading manners are based on the provisions of urban planning quota indicators [15]						
**Infrastructure Grade**						
Grade		Description				
1		Fire hydrant, plumbing well, trash can				
2		Fire hydrant, plumbing well, trash can, substation box, border tree				
3		Fire hydrant, plumbing well, trash can, substation box, border tree, streetlamp, bench				
4		Fire hydrant, plumbing well, trash can, substation box, border tree, streetlamp, bench, street art, etc.				
The above grading manners are based on the ample degrees of the current situation						

FAR, floor area ratio.

**Table 2:** Data normalization results.

Location	No.	FAR	Air Path Width (m)	Road Hierarchy	Infrastructure	Street Canyon	Building Height (m)	Average
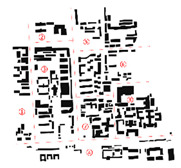	Part 1	0.00	0.00	0.00	0.00	0.00	0.00	0.00
	Part 2	0.70	0.33	0.50	0.33	0.33	0.25	0.41
	Part 3	0.90	0.33	1.00	0.67	0.33	0.25	0.58
	Part 4	0.43	0.67	1.00	0.67	0.33	0.50	0.60
	Part 5	0.55	1.00	1.00	1.00	0.33	0.50	0.73
	Part 6	1.00	1.00	1.00	1.00	1.00	1.00	1.00
	Part 7	0.85	1.00	1.00	1.00	0.67	0.75	0.88
	Part 8	0.97	1.00	0.50	0.67	0.75	0.75	0.77

## Data Availability

Not applicable.
